# Precision Medicine in Non Communicable Diseases

**DOI:** 10.22088/IJMCM.BUMS.8.2.1

**Published:** 2019-07-25

**Authors:** Mandana Hasanzad, Negar Sarhangi, Hamid Reza Aghaei Meybodi, Shekoufeh Nikfar, Fatemeh Khatami, Bagher Larijani

**Affiliations:** 1 *Medical Genomics Research Center, Tehran Medical Sciences, Islamic Azad University, Tehran, Iran.*; 2 *Personalized Medicine Research Center, Endocrinology and Metabolism Clinical Sciences Institute, Tehran University of Medical Sciences, Tehran, Iran. *; 3 *Department of Pharmacoeconomics and Pharmaceutical Administration, Faculty of Pharmacy, Tehran University of Medical Sciences, Tehran, Iran.*; 4 *Chronic Diseases Research Center, Endocrinology and Metabolism Population Sciences Institute, Tehran University of Medical Sciences, Tehran, Iran.*; 5 *Endocrinology and Metabolism Research Center, Endocrinology and Metabolism Clinical Sciences Institute, Tehran University of Medical Sciences, Tehran, Iran.*

**Keywords:** Precision medicine, non-communicable diseases, cardiovascular diseases, type 2 diabetes, chronic obstructive pulmonary disease, cancer

## Abstract

Non-communicable diseases (NCDs) are the leading cause of death and disease burden globally, cardiovascular diseases (CVDs) account for the major part of death related to NCDs followed by different types of cancer, chronic obstructive pulmonary disease (COPD), and diabetes. As the World Health Organization (WHO) and the United Nations have announced a 25% reduction in mortality of NCDs by 2025, different communities need to adopt preventive strategies for achieving this goal. Personalized medicine approach as a predictive and preventive strategy aims for a better therapeutic goal to the patients to maximize benefits and reduce harms. The clinical benefits of this approach are already realized in cancer targeted therapy, and its impact on other conditions needs more studies in different societies. In this review, we essentially describe the concept of personalized (or precision) medicine in association with NCDs and the future of precision medicine in prediction, prevention, and personalized treatment.

Non-communicable diseases (NCDs) – mainly cancers, type 2 diabetes mellitus (T2DM), cardiovascular diseases (CVDs), and chronic respiratory diseases (CRDs) are the biggest cause of death worldwide (1). More than 36 million people die annually from NCDs which account for 63% of all deaths globally (1, 2). The World Health Organization (WHO) developed an NCD global action plan and determined nine targets that have to be achieved to reach a 25% relative reduction in premature mortality from the four major NCDs (i.e., CVDs, cancers, CRDs, and diabetes) by 2025 (3).

Major advancements in basic science and publishing the human genome project data have created an opportunity for significant progress in medical practice. Hundreds of genes that harbor variations contributing to human diseases have been discovered during the last decade. 

Medicine has always tried to be as precise as possible. The terms ”personalized”, “precision”, “stratified”, “individualized” and “P4 medicine” have been used interchangeably by many clinicians and researchers (4). Precision medicine which was defined synonymously personalized medicine or individualized medicine is really not a new invention (5). In the context of precision medicine, a new taxonomy describes the common diseases based on their molecular profile in addition to traditional signs and symptoms (6). Precision medicine is an emerging approach for prediction, early prevention and treatment that takes into account individual variability in genes for each person. Precision medicine is quickly considered as the art of medicine for creating personalized assessments of health and diseases that are derived from omics (i.e., genomic, transcriptomic, proteomic, metabolomic) profile (7, 8). Precision medicine is much more well-known in cancer than in diabetes, where omics sciences help to perform the targeted therapy. 

The genetic architectures of cancer and diabetes are completely different as in genetic triggers both type 1 (T1D) and type 2 diabetes (T2D) are caused by germline variants with modest effect, whereas specific somatic mutation in a particular cancer cell type initiates cancer development (7).

## Precision type 2 diabetes medicine

Diabetes is a significant public health problem worldwide that is recognized as a considerable threat to human health (9). Hence, there is an urgent need to apply novel intervention which predicts and prevents diabetes. Evidence suggests that early treatment is crucial for the prevention of diabetes complications. Current treatment guidelines are restricted because of poor metabolic control of progressed diabetes. Although diabetes is related to insulin deficiency and elevated blood glucose, it is really a mixture of diseases and is much more heterogeneous than the present classification into type 1 and type 2.

A well- defined diabetes classification, partic-ularly for T2D could provide a robust tool to facilitate the implementation of precision medicine for a better diagnosis to a more ideal treatment approaches (10). 

In a data-driven cluster analysis in 2018, five replicable clusters of patients with diabetes, with differing disease progression and risk of diabetic complications have been identified. This new sub-stratification might finally help to tailor early treatments to patients who would benefit most, representing the first step towards precision medicine in diabetes management (11).

The American Diabetes Association (ADA) emphasizes the importance of the patient-centered approach, and moves guidelines away from a step-by-step protocol-driven.

Patient-centered care has referred to care that is respectful of and responsive to individual patient preferences, needs, and values. Therefore, patient values guide all clinical decisions, whether based on evidence or expert opinion (12). 

In the near future T2D will have been classified into a series of distinct diagnostic types (we could call them type 2A, type 2B) (13). The science and art of medicine come together in precision medicine approach when the clinician is faced with making treatment recommendations for a patient who may not meet the eligibility criteria used in the guidelines as we rely on it in evidence-based medicine approach. Accordingly, we need to look at medical practice in precision-based medicine approach manner which is an innovative approach to tailoring disease prevention and treatment that takes into account genetic make-up, environments, and lifestyles (14). Recognizing that one size does not fit all, the ADA standards provide guidance for when and how to adapt recommen-dations for an individual (12).

T2DM is the most common type of diabetes, heterogeneous, complex disease in which both genetic and environmental factors contribute to hyperglycemia which constitutes the primary hallmark of T2DM (15). Investigation for suscepti-bility genes involved in the pathophysiology of T2DM has been performed by linkage analysis, candidate gene, exome, and genome-wide associa-tion studies (GWAS) (16).

The first GWAS in T2D were published in 2007 (17). When the Human Genome Project was completed and low-cost genotyping methods brought the first successes from GWAS, there was a hope that genomics would have a remarkable impact on risk prediction, diagnosing, stratifying, preventing, and therapeutic decisions regarding genetic variability in each patient (18). GWAS have identified more than 300 loci which are robustly associated with T2DM (18).

Regardless of some GWAS that involve many hundreds of thousands of individuals and show genome-wide significance, collectively, most of the variants have modest effects on T2DM predisposi-tion and account for only 10% of overall disease risk (18-20). Several GWAS loci associated with T2DM show effect on insulin secretion pathway which is concentrated on the pancreatic islets. By the use of the other genome-based omics technologies, gene expression maps of human islets have developed, and it was demonstrated that T2DM risk variants are located in active islet enhancers. One of this important regions lies next to the melatonin receptor 1B (*MTNR1B*) gene, which encodes one of the receptors for melatonin. There is a neurogenic differentiation 1 (NeuroD1) binding site in an islet enhancer upstream of *MTNR1B* which its variation influences *MTNR1B* expression (21).

High-throughput technologies have provided comprehensive information about the genome, transcriptome, proteome, metabolome. These omics approach can help to identify subgroups of T2D which share unique biological characteristics (7). But, so far, the clinical applications of genetics in diabetes are limited to monogenic subtypes (22).

***Pharmacogenetics of type 2 diabetes***

At present, there are 12 drug classes available for T2DM management (Table 1) (12). Together with lifestyle/ behavioral therapy, metformin is generally prescribed as first-line therapy. If metformin is not well tolerated or the Hemoglobin A1C (HbA1c) goal is still not achieved after several months of treatment, a combination therapy of metformin with one or more other oral antidiabetic drugs will be initiated.

Nearly 20–40% of inter-individual differences in metabolism and response to medications are related to genetic factors (23). Pharmacogenomics studies follow major objectives such as patient stratification which try to classify patients according to their clinical response to the corresponding drug or its toxicity, or drug development by identification of the specific targets which comes from the GWAS (24). Multiple genetic polymorphisms (Table 1) have been reported for the effectiveness of oral anti-diabetic drugs, and are categorized into two groups: the classical pharmacogenomics genes affecting pharmacokinetics/pharmacodynamics, and T2DM risk genes (25-27). The recent guidelines do not consider individual variations to therapeutic response.

## Precision cardiovascular medicine

CVD remains the major leading cause of death worldwide (1). 10% of the global disease burden (DALYs) is attributed to CVD (28). CVDs are the leading cause of death worldwide, and a major barrier to human healthcare. CVD refers to all diseases of the heart and circulation system, including CVDs due to atherosclerosis; ischemic heart disease (IHD) or coronary artery disease (CAD) (e.g. heart attack), cerebrovascular disease (e.g. stroke), diseases of the aorta and arteries, including hypertension and peripheral vascular disease, and other CVDs such as coronary heart disease (CHD) known as CAD, rheumatic heart disease, cardiomyopathies and cardiac arrhythmias (28). Global progress is toward controlling lifestyle habits such as diet and smoking. But CADs are complex diseases which are caused by multiple combinations of genes and environmental factors. The heritability of CAD shave been estimated between 40% and 60% (29). CVDs are extremely preventable; investment in prevention is the most sustainable solution for the CVDs epidemic. Over the last two decades, CVDs mortality has declined in developed countries due to a combination of prevention and control measures (28). Hypertension is one of the risk factors of CVDs. Control of hypertension is an important issue to avoid deaths from CAD and stroke (30).

**Table 1:** Antidiabetic medications, related pathophysiological T2DM mechanisms and pharmacogenetically relevant target genes

**Drug**	**Pathophysiological pathway**	**Drug examples**	**Gene (s)**
**Biguanides**	Insulin signaling Inhibition of gluconeogenesis	Metformin	*SLC22A1*, S*LC22A2*, *SLC22A3*, *SLC47A1*, *SLC47A2*, *SLC29A4*, *PRKAA1*, *PRKAA2*, *STK11*, *MEF2A*, *EF2D*, *HNF1B*, *HNF4A*, *ABCC8*, *KCNJ11*, *GCK*, *CAPN10*, *ATM*, *LC2A2*, *SP1*, *AP2*, *PPARA*, *ATE2-K*, *SRR*, *TCF7L2*, *WFS1*, *ENPP1*, *TCF7L2*
**Sulfonylureas**	Increasing insulin secretion	Glyburide Gliclazide Glipizide Glimepiride Tolbutamide	*KCNJ11*, *ABCC8*, *TCF7L2*, *IRS1*, *CDKAL1*, *CDKN2A/2B*, *KCNQ1*, *CYP2C9*, *CYP2C19*, *G6PD*
**DPP4-inhibitors**	Stimulation of insulin secretion and inhibition of glucagon secretion (glucose dependent)	Sitagliptin Vildagliptin Alogliptin Linagliptin Saxagliptin	*CYP3A4*, *CYP2C8*, *TCF7L2*, *CTRB1*, *CTRB2*, *GLP-1R*, *KCNQ1*, *PRKD1*, *CNTN3*, *ASK*, *OC10537792*
**Meglitinides**	Enhancement of insulin secretion	Repaglinide Nateglinide	*SLCO1B1*, *OATP1B1*, *CYP2C9*, *CYP2C8*, *CYP3A4*, *PAX4*, *NEUROD1*/*BETA2*, *KCNJ11*, *SLC30A8*, *NAMPT*, *OS1AP*, *UCP2*, *KCNQ1*, *TCF7L2*, *IGFBP2*, *MDR1*, *PAX4*
**Thiazolidinediones**	Insulin sensitization	Pioglitazone Rosiglitazone Troglitazone Ciglitazone	*PPARG2*, *ADIPOQ1*, *CYP2C8*, *CYP2C9*, *CYP3A4*, *PTPRD*, *ACE*, *SLC30A8*, *ABCA1*, *KCNQ1*, *RBP4*, *MTHFR*
**SGLT2 inhibitors**	Renal glucose excretion	Canagliflozin Dapagliflozin Empagliflozin	No gene with relevant effects on treatment response was described
**α-glucosidase** **inhibitors**	Inhibition of glucose absorption by inhibition of intestinal glucosidase	Acarbose Miglitol Voglibose	*PPARG*, *PPARA*, *PPARGC1A*, *HNF4A*, *LIPC*
**Glucagon-like** **peptide-1(GLP-1)** **receptor agonists**	Increase of glucose stimulated insulin secretion, functional pancreatic β-cell mass and decrease ofglucagon secretion from pancreatic α-cells	Exenatide Albiglutide Dulaglutide Liraglutide Lixisenatide	*GLP-1 R*, *CNR1*
**Insulin**	Increasingglucose disposal and decreasing hepatic glucose production	Lispro Aspart Glulisine Inhaled insulin Human Regular Human NPH Glargine Detemir Degludec Basal insulin peglispro	*GLP1R*, COMT, *DBH*, *PNPLA3*, *TM6SF2*, *ACE*
**Bile acid seqestrants**	Glucose lowering (is not known) Decrease in hepatic glucose production (HGP)	Colesevelam	No gene with relevant effects on treatment response was described
**Dopamine-2 agonists**	Increasing insulin sensitivity and modulatinghypothalamic regulation of metabolism	Bromocriptine	No gene with relevant effects on treatment response was described
**Amylin mimetrics**	Slows gastric emptying, promotingsatiety and reducingthe postprandial glucagons increase	Pramlintide	No gene with relevant effects on treatment response was described

Modern “omics” approach applies for cardiovascular precision medicine. Since 2007, GWASs have shown a great contribution to the identification of genetic loci of many multifactorial diseases. The first GWASs of CAD were reported in 2007, and an association between common single nucleotide polymorphisms (SNPs) on chromosome 9p21 and CAD was confirmed; this locus still has shown the strongest known association with CAD (31). Common variants in chromosome 9p21 were associated with CAD (29).

Several GWAS have been performed for CADs and near 163 loci have now been associated with CAD at a genome-wide level of significance (32). The present approach to decreasing cardiovascular morbidity and mortality in a high-risk individual is based on evidence-based medicine. For decades, the approach to clinical care has involved evidence-based medicine, which is the appraisal of a small set of a patient’s symptoms and “one size fits all” therapeutic approach. In this system, there is no attention to individual variations which is concentrated in the etiology and pathophysiology of disease (33). New advances in DNA technology and omics approaches (genomics, transcriptomics, and proteomics) have indicated an enormous awareness that conventional medicine approach cannot consider all of the contributors to the etiology of CVD. The power of precision medicine is influenced by the big data that arise from omics approach, and also combining their data sets to standard clinical exams. The evolution of CVD precision medicine revolutionizes health care in prediction, prevention, and tailored treatment options which enter into reality in the field of oncology (34).

The difference between precision medicine in CVD with other conditions is that CVDs are the consequences of many chronic problems such as obesity, diabetes, and hypertension.

As a simple example consider individuals with blood pressure (BP) or blood cholesterol levels, the choice of antihypertensive therapy or cholesterol lipid-lowering drugs depends on the genomic profile of each individual that should be taken into account by clinicians. Regarding genetic architecture of hypertension, near 40 genes have been identified that each influences BP with small effect (only 1 mm Hg), so potentially hundreds of gene variants may have fine effects on BP (35). Hence, a number of genetic variants have been linked to hypertension and CAD, but their effect sizes are small too (36).

The majority of recognized risk alleles were not a causative variant, but likely they are nearby a causative variant (32). Novel target therapy based on proprotein convertase subtilisin/ kexin type 9 (*PCSK9*), guanylate cyclase 1 soluble subunit alpha 1 (*GUCY1A1*), angiopoietin-like 3 and 4 (*ANGPTL3*, *ANGPTL4*) is proposed by CAD GWAS research (32).

The other face of CVD personalized medicine is CVD stratification (e.g., CAD, heart failure, CHD, etc.) which is the identification of a group of patients who will benefit from a specific intervention in tailored treatment (37). Several fundamental classes of CVD medications including β-adrenergic receptor blockers, lipid-lowering drugs, antithrombotic agents and angiotensin-converting enzyme inhibitors have been introduced in CVD pharmacogenomics (38). 

CVD precision medicine has the potential to change conventional standards of care, although acceptance of precision medicine will require some evidence for its effectiveness as an approach to care. Clinicians need to know that the data from the omics approach will lead to an actionable step that transforms the routine treatment options.

## Precision chronic obstructive pulmonary disease medicine

Chronic obstructive pulmonary disease (COPD) is a common pulmonary disease which is influenced by a set of complex interactions between the genetic and environmental factors. COPD is one of the five causes of morbidity and mortality all over the world, and is usually characterized by airflow limitation, airway remodeling, and chronic inflammation (39-41). COPD is a condition with molecular and cellular extensive modifications which results in similar clinical symptoms like cough, dyspnea, and wheeze (42). Global initiative for obstructive lung disease (GOLD) therapeutic strategy for COPD was suggested based on evaluating the severity of disease and therapeutic decisions in the past (39, 43). So, there was no beneficial suggestion validated except persuading the patient to quit smoking and taking some short-acting bronchodilators and/or theophylline although it was unfortunately associated with significant side effects (43). Due to the progress of our recent knowledge of COPD over the past two decades, we know a lot about COPD as a complex and heterogeneous condition with multiple phenotypes and endotypes (43).

The complexity of COPD indicated that a large number of intrapulmonary and extrapulmonary components with non-linear dynamic interactions exist in COPD whereas, heterogeneity revealed that not all of these components are presented in all individuals at once (39). Therefore, understanding the genetic architecture of COPD may be applicable in clinical practice, which deals with individual patients personalized medicine contrary to the groups of patients classified according to a particular clinical phenotype (43). It means that there is an extreme need for COPD care to change from the current “one-size-fits-all” approach to precision medicine in the way of individual variability in genes, environment, and lifestyle for each patient (44). The precision medicine initiative created from 2015, has brought new insight on precision medicine approaches for the management of a wide range of disease including COPD (39). Precision medicine is supported by biomarkers to recognize subgroups of patients who will most be expected to benefit from therapeutics decisions and those who will only experience harm (predictive biomarkers); predict therapeutic responses to drugs at an individual level (response biomarkers) and segregate patients who are at risk of poor outcomes from those who have relatively stable disease (prognostic biomarkers) (44). In the field of both COPD cause and appearance, precision medicine approaches improve assessment, treatment, and outcomes (42). More than recent advances of precision medicine in the current management status of COPD, the future of COPD will certainly become even more personalized. It is expected that precision medicine will offer a better opportunity for COPD in the way of using predictive biomarkers in COPD manage-ment (45, 46).

Precision medicine progress on COPD requires identification of related endotypes, the basis of COPD pathobiological mechanism, with the identification of phenotypes complicated in the complex and heterogeneous appearance of the disease (40).

## Precision cancer medicine

**Fig. 1:**
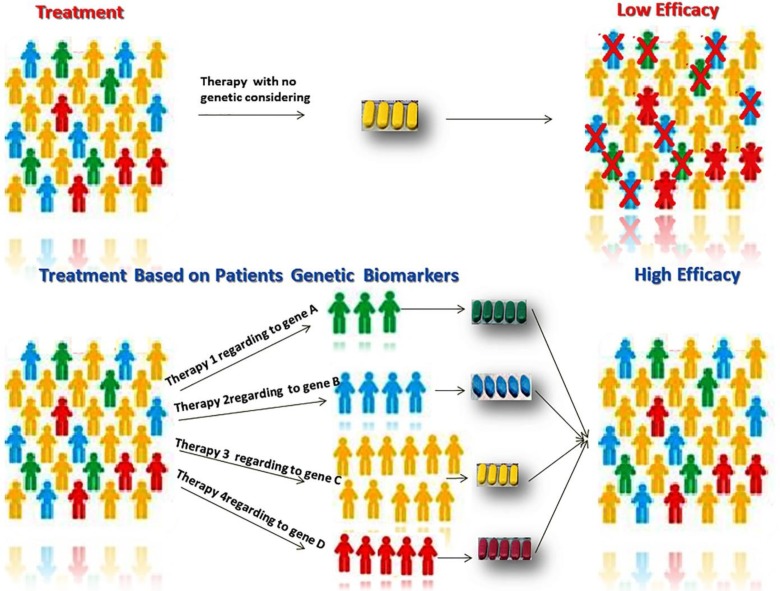
Higher efficacy of cancer therapy is possible if patients are categorized based on their genomics’ data

Cancer is an important problem worldwide and is the second leading cause of death globally. According to Global cancer statistics, cancer is accountable for an expected 9.6 million deaths in 2018 (47, 48). The most common causes of cancer death are related to six cancers including lung (1.76 million deaths), colorectal (862 000 deaths), stomach (783 000 deaths), liver (782 000 deaths), breast (627 000 deaths), and prostate (29, 49). In addition, thyroid cancer is a cancer with different biological behaviors and prognostic factors (50). After the completion of human genome project, considerable progress has been made in knowing the genomics of tumor which has been applied in personalized/ precision medicine clinical practice (51). In fact, targeted therapies have been developed in oncology and brought the new idea of cancer therapeutic approach with the maximum effect (Figure 1). Following, we are reviewing the precision medicine of the most common cancer types. Following the precision medicine development, the predictive models have been created decision support systems (DSSs) which help radiation and clinical oncologists to make a better decision (52).

***Precision lung cancer medicine***

Lung cancer is the most common form of cancer with a high mortality rate in both genders all over the world. The furthermost subtype of lung cancer is the non-small cell lung cancer (NSCLC) which made 85% of lung cancers with the 5-year survival rate less than 15% (53). Treatment choices based mainly on the stage (extent) of cancer and radical surgery are the usual therapy choices for NSCLC stages I through IIIA patients (54). After that, in all resected cases excluding stage IA, the treatment strategy is based on adjuvant chemotherapy after surgical resection (55). For patients in stage II and IIIA adjuvant cisplatin-based chemotherapy is suggested as the gold standard for totally resected NSCLC tumors that reduced the risk of local relapse and non-brain metastasis (56). Radiotherapy must be undertaken in patients with two number of metastatic (N2) lymph nodes as well. In advanced stage IIIB/IV or inoperable NSCLC patients, multidisciplinary management must be performed with four cycles of cisplatin-based chemotherapy over a third generation cytotoxic agent or a cytostatic (anti-epidermal growth factor receptor (EGFR), anti-vascular epithelial growth factor Reseptor (VEGFR) drug (57). Before the precision medicine approach in cancer therapy, any NSCLC patient was treated by chemotherapy without histology or any other genetic biomarker consideration. The new aspect of precision medicine has developed the incorporation of genomics tumor data into candidate patient’s diagnosis with the lowest treatment toxicity and highest treatment benefit as well (58). The anti-cancer agents such as anti-EGFR and tyrosine kinase inhibitors (TKIs) are well-known medicine for patients with advanced NSCLC (59). There are different groups of TKIs (gefitinib, erlotinib, afatinib, neratinib, and the most recent one osimertinib) that their efficacy is dependent on overexpression of *EGFR* or Kirsten rat sarcoma 2 viral oncogene homolog* (KRAS) *like (60). Due to the blossoming of the liquid biopsy approach in order to track the tumor genetic via plasma of cancer patients, now it is possible to determine the circulating *EGFR* mutation. The first U.S. Food and Drug Administration (FDA) approved liquid biopsy test is based on circulating- free tumor DNA *EGFR* mutation in personalized treatment of NSCLC (61). Additionally, it was shown that the combination of exosomal RNA (exoRNA) and cell-free DNA (cfDNA) enhanced the sensitivity of liquid biopsy for *EGFR* mutation detection in NSCLC versus standard circulating tumor DNA (ctDNA) alone (62). 

Another molecular pathway triggering lung tumorgenesis is the fusion of the anaplastic lymphoma kinase (*ALK*) with echinoderm microtubule- associated protein like 4 (*EML-4*) that normally are essential for proper neuronal development and microtubule formation (63). Crizotinib is an ALK-inhibitor targeting EML4-ALK fusion protein that suppresses its carcinogenic kinase activity (64).

The other NSCLC therapy strategy is based on monoclonal antibodies approving by US FDA which classically target the interaction between the *programmed death-ligand 1* (*PD-L1*) and the *programmed cell death protein 1 (PD-1*) receptor (65). Atezolizumab, nivolumab, and pembro-lizumab are targeting the PD-L1 ligand and receptor (66).To predict the precise efficacy of pembrolizumab in NSCLC patients, it is important to determine the PD-L1 protein levels, as well as *CD8, Janus kinase 1* *(JAK1), Janus kinase 2 (JAK2), β2 microglobulin (B2M) *expression. Zaretsky's study on pembrolizumab -resistant patient models presented that inactivating mutations in* JAK1, JAK2*, and *B2M*can converse drug resistance in NSCLC patients (67).

***Precision colorectal cancer medicine***

Colorectal cancer (CRC) is the colon or the rectum cancer that is also called “colon cancer” or “rectal cancer” as well. CRC is the third most frequent cancer in men and the second most common one in women (68). CRC screening is an essential process for detecting early-stage CRCs in suspicious cases with positive family history and aged subjects. Nevertheless, more than 80% of CRC cases have no CRC family history (69). The very recent recommendation is determining personalized CRC screening starting age based on lifestyle, environmental, and genetic factors. This mode was developed by Jeon et al. as the risk prediction models for CRC based on 19 lifestyle and environmental factors and 63 mutual genetic variants recognized to be linked to the CRC risk using data from 14 population-based studies (70). In addition, the higher plasma glypican-1 positive (GPC1+) exosomes and less miR-96-5p and miR-149 expression can be specific indicators of CRC diagnosis (71).

Regarding CRC treatment, two central molecular mechanisms are described in CRC including 85% with chromosomal instability (CIN) and 15% with microsatellite instability (MSI) (72). The knowledge of genomics has changed molecular markers for precision CRC diagnosis and treatment. In fact, large inter and intra tumor heterogeneity of CRC can be the main reason for treatment failure (73). Molecular testing should be done in medical practice to select the targeted biological agents and determining pharmacoresistance in CRC toward personalized drug toxicity and efficacy. There is an established progress in response rate and patient's survival through targeting therapy by anti- EGFR monoclonal antibodies cetuximab and panitumumab, bevacizumab, aflibercept, and regorafenib in combination with typical fluor-opyrimidines- based chemotherapeutic regimens (74). Moreover, the methylenetetrahydrofolate reductase (MTHFR) -1298 A>C (rs1801131) polymorphism can be a good predictor of survival in stage II/III CRC patients in response to adjuvant fluoropyrimidine chemotherapy with or without oxaliplatin(75).

Inherited genetic alterations of ATP-binding cassette (ABC) and solute carrier (SLC) drug transporter, as well as EGF and VEGF signaling pathways, have been linked to the individual tumor sensitivity phenotype in CRC patients who are under treatment of fluoropyrimidines combined with each irinotecan or oxaliplatin (76). Nowadays, computation of clinical- pharmacogenetic algorithms, conjoining several SNPs with clinico- demographic landscapes, represents a consistent approach of predicting tumor response to therapy (77). Polymorphisms of *xeroderma pigmentosum complementation group D* (*XPD*) and dihydropyrimidine dehydrogenase (*DPYD*) coding genes is a chief prognostic factor in the therapeutic approach of CRC with platinum drugs and 5-fluorouracil (78). Survivin as an inhibitor of apoptosis (IAP) protein family that inhibits caspases and blocks cell death, might be a self-determining prognostic element and a suitable target for the chemoradiotherapy of CRC patients (79).

***Precision stomach/gastric cancer medicine***

Stomach cancer is considered as an aggressive, uncontrolled growth of stomach cells which is also called gastric cancer. Stomach cancer is a highly fatal malignancy and is the third leading cause of death from cancer worldwide (80). Diagnosis of stomach cancer is one of the problematic issues because conventional diagnostic methods are not as specified and sensitive as they should be. Very recently the knowledge of biosensor has brought the promising direction in early diagnosis of stomach cancer and its personalized management (81). In fact, the major component of liquid biopsy which is circulating tumor cells (CTCs) can provide a suitable target for cancer diagnosis through nano biosensor detection system. CTCs with their stem cell-like properties can be a crucial marker of gastric cancer stem cells (82). The existence of CTCs in the peripheral blood suggested a complete powerful link with the occurrence of metastasis and secondary tumor formation in other tissues (83). Thus, the presence of CTCs or even one cell per 10 ml of blood can be the indicator of poor prognosis (84). CTCs enumeration and molecular characterization can be recruited for personalized stomach cancer diagnosis (85). Based on several studies related to the clinical impact of CTC, the presence of CTC can be the poor prognosis indicator in patients with stomach cancer (86). CTCs can be an alternate marker for determining response to chemotherapy in patients with advanced stomach cancer (87). A new classification system according to MSI and gene expression profile is presented by Asian Cancer Research Group across multiple stomach cohorts. There are four main molecular subtypes of stomach cancer including MSI, microsatellite stable with epithelial- to- mesenchymal transition features (MSS/EMT), MSS/TP53 mutant (MSS/TP53^+^), and MSS/TP53 wild-type (MSS/TP53^–^) (88). Distinct subtypes of stomach cancer were categorized by molecular characterization including novel mutational signatures and hypermethylated regions with prognostic capability (89).

The surgery plan does not usually afford a complete cure, even in the early stages of disease, and tumor recurrence usually happens (90). Chemotherapy, multimodality therapy and target therapy have revealed specific benefit in the treatment of gastric cancer. Herceptin® (trastuzumab), a monoclonal antibody against human epidermal growth factor receptor 2 (HER2), given with platinum-based chemotherapy is the typical first-line regimen in HER2-positive advanced stomach cancer but there is no standard approach in the second-line setting (79). While numerous new agents are still being investigated for targeted stomach cancer therapy, some current clinical trials are now targeting *STAT3*, *c-MET*, *mTOR*, *CLDN18.2,* and *PD-1/PD-L1 *(91). There is a multicenter phase 1b trial of pembrolizumab (KEYNOTE-012), which presented strong decreases in a subset of patients with PD-L1–positive advanced stomach cancer identified by a prototype assay technique (92).There are some investigations over epigenetic targeting in cancer therapy in which combination therapy with vorinostat (a histone deacetylase inhibitor) and radiotherapy is evaluated in gastrointestinal cancer.

***Precision liver cancer medicine***

Liver cancer (hepatic cancer) is a cancer that starts from the liver cells or has spread from elsewhere to the liver, known as liver metastasis (93). This cancer has the 5-year survival rate of about 18% which is accounting for approximately 41,000 cancer cases and 29,000 deaths in the United States in 2017 (53, 94). Although percutaneous liver biopsy is still a standard diagnostic procedure, it has serious complications such as bleeding after biopsy (95). Very recent reports indicated that next- generation sequencing of cfDNA can offer a therapeutically actionable genomic analysis in hepatocellular carcinoma (HCC) (96). In fact, liver cancer is a highly heterogeneous cancer and this heterogeneity can be modified and refined to diagnose and treat patients in a personalized manner. HCC diagnosis is possible without pathologic confirmation by measuring the serum alpha-fetoprotein (AFP) level together with imaging techniques, including ultrasonography, magnetic resonance imaging, and computerized tomography (97). Primary liver cancer includes HCC, intrahepatic cholangiocar-cinoma (iCCA), and other rare tumors, especially fibrolamellar carcinoma and hepatoblastoma (98).

Finding new therapeutic targets centered on the molecular pathways that are involved in liver carcinogenesis have directed in the targeted treatment of HCC patients (99). TKIs have the excessive potential of HCC therapy over targeting several growth factors and their associated signaling pathways like EGF/EGFR, VEGF/ VEGFR, IGF/IGFR, PDGF, FGF, RAS/ RAF/ERK/MAPK, PI3K/AKT/ mTOR, Wnt/ beta-catenin (100). Now, there are about sixty investigated components for treatment of HCC, but only sorafenib that targets both Raf, VEGF and PDGF receptor tyrosine kinase signaling has suggested effective results in patients with advanced HCC (101). Other TKIs like sunitinib, linifanib, brivanib, and regorafenib suppress a number of angiogenesis-related signaling pathways, such as VEGFR, PDGFR, and FGFR (102). Even though numerous clinical trials have been stopped because of underprivileged efficiency or severe adverse effects, these lines shed a light on the mechanisms of targeted therapy for HCC and can lastly make it possible to optimize the current therapies for this fatal disease in the way of precision medicine.

***Precision breast cancer mMedicine***

Breast cancer is the most prevalent universal cancer type among women. About 1.6 million new cases of breast cancer were reported in 2012 which caused more than 500,000 deaths (103). According to certain breast cancer-associated biomarkers like estrogen receptor (ER), progesterone receptor (PR), Ki-67 (a protein marker with prognostic and predictive potential for adjuvant chemotherapy), and human epidermal growth factor receptor 2 (HER2), breast cancer is classified to the luminal A, luminal B, HER2-positive, and triple-negative (104). In spite of the fact that the improvement of precision medicine in breast cancer has mostly increased the overall survival of patients, there are still some barriers for personalized treatment of breast cancer patients that result in dissimilarities in the level of responses to different cancer treatment regimens (chemotherapy, radiation, or surgical treatments) (105).

In premature menopause, aged 40 or below, the estrogen production decreases, so some SNPs sinvolved in the metabolism of estrogen can give information about breast cancer targeting therapy in premature menopause or chemotherapy-induced menopause in breast cancer patients (106-108). A study in the Brazilian population revealed that polymorphisms in the genes coding for estrogen receptors (*ESR1* and *ESR2*) were connected to the premature ovarian failure, a feature of premature menopause (109). Chemotherapy in patients with amenorrhea showed better outcomes and increased overall survival and progression- free survival rates (110).

Detecting the point mutation of protein kinase B (*PKB/AKT1*)(*AKT1E17K* mutation) in both tissue and plasma samples of advanced breast cancer patients can help for decision making in treatment approach by rapamycin in order to inhibit tumor progression (77).

HER2 is considered usually for selecting chemotherapeutic drugs such as trastuzumab that target this protein. There has been some recent evidence that HER2 expression can be altered in several stages across the cancer trajectory, so trastuzumab therapy cannot be centered completely on the HER2 in primary tumors (111). The mitochondrial DNA (mtDNA) common deletions are the common genetic alterations that happen in breast cancer patients (112). Some mtDNA insertion/deletion can have a critical role in chemotherapeutic drug resistance and treatment results (113). 

***Precision prostate cancer medicine***

Prostate cancer is one of the most common types of cancer in men and is the most common cause of global death, accounting for an estimated 366,000 deaths and 6.3 million disability-adjusted life years in 2015 (114). An incident case of prostate cancer has increased over the last fifteen years (115). Heterogeneous genomic aberrations could have a consequence in prostate cancer onset, progression and metastasis, and different drug responses that can be observed between individual patients. There are some transcription factors involved in androgen receptor gene expression regulation like Forkhead Box A1, GATA-binding protein 2 and octamer- binding protein 1 (116). In addition, transmembrane serine protease (TMPRSS2) is the prostate- specific protein. The transcriptional regulator Erg (ERG)-TMPRSS2, speckled-type POZ protein (SPOP), tumor protein 53 (TP53), phosphatase and tensin homolog (PTEN), ataxia telangiectasia mutated (ATM) and catenin β1 are the most frequently mutated cancer-driving genes of prostate cancer (117). There is a large family member of transcription factors involved in different tissue development as well as cancer progression that are known as erythroblast transformation-specific (ETS). This family is shown as the fusion form of ERG- transmembrance protease serine 2 *(ERG-TMRPSS2) *in the prostate cancer*.* For *ETS* fusion-positive cancer, agents inhibiting fusion cofactors like poly ADP-ribose and histone deacetylase can have an impact on patients who are carriers of this fusion (118). Rapamycin is the drug that targets (PI3K/Akt / mTOR) signaling pathway, so can be the best choice for the patients with *PTEN* loss or mutation. FISH studies of *PTEN* gene loss and *ERG/ETV1* gene rearrangements could be taken into the account of prostate patient management (119). Mutations in DNA repair genes like *BRCA2* and *ATM *can have resulted in higher sensitivity to treatment with platinum agents or poly ADP ribose polymerase inhibitors (120).

Some long noncoding RNAs (lncRNAs) have been recognized in prostate tissue and some studies indicated their changed expression pattern during prostate tumorigenesis (121). More than genetic alterations some epigenetic changes are suggested as the prostate cancer hallmarks, including genomic global hypermethylation (122). The androgen receptor is overexpressed and hyperactivated in human castration-resistant prostate cancer (CRPC). It was shown that RAR-related orphan receptor gamma (ROR-γ) is a crucial factor in CRPC through androgen receptor pathway, and is considered as a potential therapeutic target for advanced prostate cancer (123). Over-expression of androgen receptor and cytoplasmic *CYP17* together can develop a response to abiraterone and enzalutamide especially in patients with bone metastasis (124). Cancer with high expression levels of the drug efflux transporter genes, including *multidrug resistance protein 1* (*MDR1*) and *certain β-tubulin isotypes* (βIII-tubulin) demonstrate increased resistance to chemotherapies, such as docetaxel (125). Reduction of the intracellular docetaxel through the high substrate affinity of MDR1 or altered microtubule binding structure by the isotype βIII-tubulin, contributes to taxane resistance. A previous archival cohort study reported that patients with down-regulated E-cadherin were associated with poor relapse outcomes following radiation therapy (126).

***Precision thyroid cancer medicine***

Thyroid is a butterfly-shaped gland in the neck, just above the collarbone that makes hormones that support the body work normally. There are several types of cancer of the thyroid gland including papillary thyroid carcinoma (PTC), follicular thyroid carcinoma (FTC), anaplastic thyroid carcinoma (ATC), and medullary thyroid carcinoma (MTC) that arises from thyroid parafollicular (C) cells (127). It is predicted that thyroid cancer will take the place of colorectal cancer as the fourth leading cancer diagnosis by 2030 (128). The most important molecular mechanism in thyroid tumorigenesis is the Ras- Raf-MEK-MAP-ERK kinase signaling pathway in the development of both PTC and FTC. In PTC, activating mutations in the gene encoding the serine/threonine kinase *BRAF*, and RET tyrosine kinase rearrangements (*RET/PTC* oncogenes) can make constitutive activation of this cascade, resulting in the thyroid cancer in most populations (129). Recently some types of multitargeted kinase inhibitors have been offered for patients with advanced or progressing metastatic thyroid cancers, and indicated higher response rates in comparison with cytotoxic chemotherapy (130). Some circulating cf DNA genetic and epigenetic alterations can be taken into the account of targeted thyroid cancer treatment (131). In 2016 a non- randomised, multicenter, open-label, phase 2 trial by Brose et.al. indicated that vemurafenib is effective in patients with *BRAF*V600E-positive metastatic or unresectable papillary thyroid cancer refractory to radioactive iodine who had never received TKI therapy (132).

The worldwide epidemic of NCDs causes some challenges to the health systems of all countries, especially in low- and middle- income countries. Prevention of premature death due to NCDs is one of the main goals of health policy. So improving the early diagnosis, prevention and treatment of NCDs and their related complications will be the principal goals in clinical practice. In precision medicine, the translation of basic findings (omics approach) in fundamental research into medical practice and meaningful health outcome will happen. Precision medicine is ready to become the biggest revolution in the practice of medicine.

Precision medicine changes standard practice and draws from clinical testing, big data sets, and systems biology in order to create an individual- specific phenotype which consequently recognize the best intervention with minimal risk. But an urgent question is whether we are ready for precision medicine in NCDs. 

## Conflict of interest

Author declare no conflict of interest.
